# The modifying role of physical activity in the cross-sectional and longitudinal association of health-related quality of life with physiological functioning-based latent classes and metabolic syndrome

**DOI:** 10.1186/s12955-020-01557-z

**Published:** 2020-10-20

**Authors:** Paco Cerletti, Dirk Keidel, Medea Imboden, Christian Schindler, Nicole Probst-Hensch

**Affiliations:** 1 Swiss Tropical and Public Health Institute, Socinstrasse 57, 4051 Basel, CH Switzerland; 2 University of Basel, Petersplatz 1, 4001 Basel, CH Switzerland

**Keywords:** Latent class analysis, Aging, Lifestyle, Physical activity, Cardio-metabolic, Metabolic syndrome, Physiological functioning, Health-related quality of life

## Abstract

**Background:**

Single cardio-metabolic risk factors are each known modifiable risk factors for adverse health and quality of life outcomes. Yet, evidence on the clustered effect of these parameters and the metabolic syndrome (MetS) on health-related quality of life (HRQoL) is still limited and mostly cross-sectional. The objectives of this study were to identify clusters of cardio-metabolic physiological functioning, to assess their associations with HRQoL in comparison with the MetS, to elucidate the modifying role of physical activity, and to assess differences in health service utilization.

**Methods:**

This study is based on longitudinal data from two time points (2010/11 & 2017/18) of the Swiss Study on Air Pollution and Lung and Heart Diseases (SAPALDIA). Latent class analysis (LCA) grouped participants based on a priori selected cardio-metabolic and MetS related physiological functioning variables *(*Body mass index, body fat, glycated hemoglobin, blood triglycerides, blood pressure). The 36-item Short-Form Health Survey (SF-36) was used to assess HRQoL. Quantile regressions were performed with and without adjustment for physical activity, to detect independent associations of the latent classes, MetS and physical activity with HRQoL. To assess the modifying role of physical activity, we additionally grouped participants based on the combination of physical activity and latent classes or MetS, respectively. Logistic regressions were used to investigate health service utilization as outcome.

**Results:**

The LCA resulted in three classes labeled “Healthy” (30% of participants in 2017/18), “At risk” and “Unhealthy” (29%). The Unhealthy class scored lowest in all physical component scores of HRQoL. Compared to healthy and active participants, inactive participants in the “Unhealthy” class showed lower scores in the physical functioning domain both cross-sectionally (− 9.10 (− 12.02; − 6.18)) and longitudinally. This group had an odds ratio of 2.69 (1.52; 4.74) for being hospitalized in the previous 12 months.

**Conclusions:**

These results point to subjects with adverse cardio-metabolic physiological functioning and low activity levels as an important target group for health promotion and maintenance of well-being. The promotion of physical activity at the early stages of aging seems pivotal to mitigate the impact of the MetS on HRQoL at higher age.

## Introduction

In the Western world, the proportion of people aged 80 years and above is estimated to double by 2080 compared to 2014 [1]. At older age disability adjusted life years and years lived with disabilities are increasing considerably [2, 3]. At the same time reduced quality of life (QoL) has been associated with older age [4, 5]. This emphasizes the global importance in investing into healthy aging and the maintenance of QoL. The concept of QoL is characterized by high complexity involving as well as influencing several life domains. In the last decades, the notion of health-related quality of life (HRQoL) has emerged [6] and is on the individual level an expression of physical and mental health perceptions.

Previous studies have commonly investigated effects of lifestyle and physiological functioning parameters on HRQoL domains one factor at a time. Yet, the simultaneous examination of these risk factors, such as combinations of cardio-metabolic physiological functioning and physical activity, provides valuable insights for possible improvements in population health and HRQoL [7, 8]. With advancing age, the physiology of numerous organ systems changes significantly. These continuous alterations lead to a decrease in several functions. The most often observed changes are increasing body fat and loss of muscle mass that commonly result in physical inactivity and vice versa [9].

A clustering approach is frequently used to examine the possible synergy of several factors and to give insights on important underlying patterns, e.g. related to physiological functioning. Several clustering methods (model and non-model-based) are available for this purpose. Latent Class Analysis (LCA) is a model-based approach that attempts to detect homogeneous groups within a heterogeneous population. It is a sophisticated tool to capture the complexity of interrelated risk factors [10]. Three recent studies and one systematic-review looked at the association of lifestyle behaviors and physiological functioning with HRQoL domains [7, 11, 12].

Furthermore, the metabolic syndrome (MetS) is a good example for the importance of investigating effects of multiple predictors with one health outcome. In particular, components of the metabolic syndrome, i.e. central obesity, hyperglycemia, dyslipidemia, and hypertension, and thus lifestyle factors related to the MetS are prevalent early warning signs in aging populations and increase the risk of a range of common chronic cerebrovascular, cardiovascular, and neurological disorders known to be associated with severe disability [13]. As shown in a systematic review, the MetS points towards worsening HRQoL, even though evidence is still limited and primarily derived from cross-sectional studies [14].

Whether physical activity can rescue persons with MetS from poor quality of life remains poorly understood with some limited suggestive evidence [15–17]. But physical inactivity has in itself been associated with both, poor HRQoL and cardiovascular morbidity. According to a UK biobank study, physical inactivity significantly increased the all-cause mortality and cardiovascular disease mortality in middle- to old- aged subjects [18], which was also seen in a study of the Kadoorie biobank [19]. Besides its direct effects on cardiovascular phenotypes such as hypertension, physical activity may additionally lower the need for medication and thereby prevent well-known side effects, which often affect HRQoL [20]. Especially in older adults physical activity can thus have major benefits for HRQoL [21].

However, none of the mentioned studies looked at the association of the identified clusters in combination with physical activity status with HRQoL domains in a longitudinal population-based setting with nation-wide coverage. Consequently, there is a research gap in assessing the linked patterns of cardio-metabolic physiological factors and physical activity – being the most prevalent lifestyle variable - with associations of HRQoL.

To enhance the understanding of joint cross-sectional and longitudinal associations of physiological functioning and physical activity with HRQoL in the general population, the objectives of this study were (1) to identify common latent classes of physiological functioning related to cardio-metabolic health in the Swiss Study on Air Pollution and Lung and Heart Diseases (SAPALDIA), (2) to assess associations of the identified latent classes with HRQoL and compare them to MetS-HRQoL associations, (3) to identify possible protective influences of physical activity in these associations and finally (4) to elucidate differences in health service utilization between the latent classes and persons with and without MetS.

## Methods

### Study population

SAPALDIA is a population-based cohort with associated biobank initiated in 1991. In SAPALDIA1, 9′651 adults (18–62 years) were randomly recruited from eight study areas in Switzerland representing the country’s geographic and cultural diversity [22]. In the subsequent decades, two follow-ups were carried out including 8′047 subjects in SAPALDIA2 (2001/2002) and 6′088 in SAPALDIA3 (2010/2011) [23]. All three assessments comprised questionnaires (based on validated instruments as for example IPAQ for physical activity) and health examinations of increasing complexity over time.

The current research is based on the subsample of participants of the third follow-up (SAPALDIA4, 2017/18), who were aged 55 years and older at the time. Unlike previous SAPALDIA surveys, data collection in SAPALDIA4 was primarily questionnaire based. Only SAPALDIA participants who were aged 55 years and older and who had answered all SAPALDIA4 questionnaires were subsequently invited for a health assessment (SAPALDIA55+) to one of the eight local study centers (Aarau, Basel, Davos, Geneva, Lugano, Montana, Payerne and Wald). The SAPALDIA55+ health visit focused on the collection of healthy aging related determinants and preclinical aging endpoints. SAPALDIA4 involved a total of 5′149 participants answering multiple self-administered questionnaires. The subgroup of participants aged 55 years and older who underwent a health examination and answered additional age-related questions consisted of *n* = 1′746 participants. While cross-sectional analyses were restricted to SAPALDIA4 participants 55+, we used data of the same subjects from the prior follow-up (SAPALDIA3) to assess longitudinal associations.

**Fig. 1:**
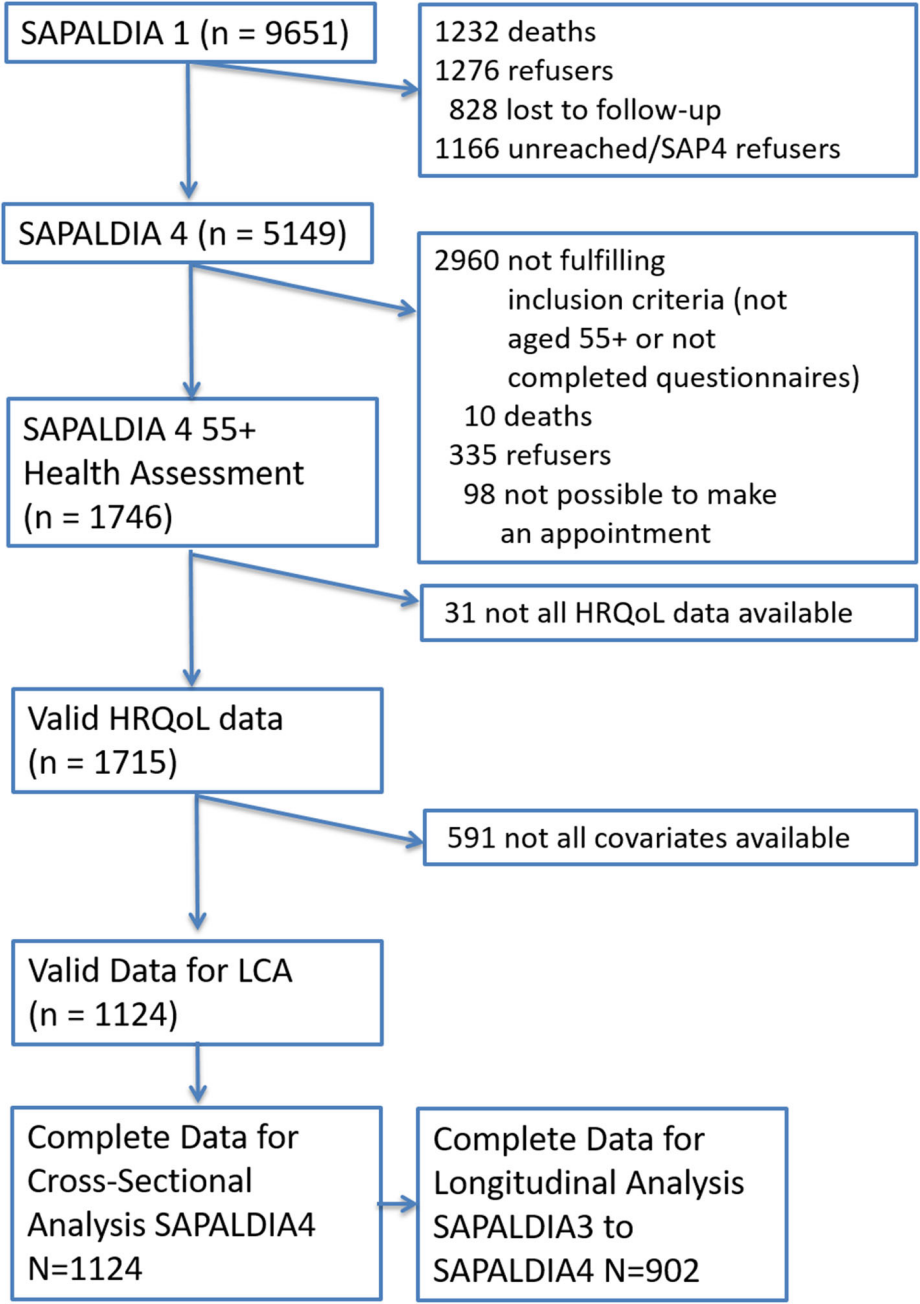
Participation from SAPALDIA 1 to the current study (1991–2018)

The present analysis ultimately involved 1′124 of the SAPALDIA4 55+ participants who provided complete information on all relevant variables. For 902 of these subjects complete information was also available from their SAPALDIA3 assessment for longitudinal analyses (Fig. 1). To assess bias due to loss-of-follow-up, the baseline characteristics at SAPALDIA1 of participants who reached the age of 55+ at the time of the health assessments, stratified by participation status at SAPALDIA4 55+ examination, can be seen in Additional Table 1.

The SAPALDIA cohort study complies with the Declaration of Helsinki. For each survey, ethics approval was granted by the regional ethics committees and participants provided written informed consent prior to participation.

### Questionnaire derived information

For SAPALDIA4, cohort participants were invited to answer multiple self-administered questionnaires on paper or online. The questionnaires consisted of information on socio-demographic characteristics, lifestyle, psychosocial factors, disease symptoms, diagnoses, medications, as well as health and social service use. The SAPALDIA4 55+ assessment additionally included a questionnaire specifically addressing aging-related determinants and risk factors.

In contrast to the self-administration of SAPALDIA4 questionnaires, SAPALDIA3 questionnaires were applied in the context of in-person interviews. Relevant questionnaire information for this analysis were questions on physical activity levels (frequency of moderate and vigorous physical activity according to WHO guidelines) as well as the covariates: sex, age, and education level (Low = Primary School (≤ 9 years), Middle = Secondary school, middle school or apprenticeship (≤12 years), High = Technical College or University (≥12 years)). We chose for this population, education levels as a proxy for socio-economic status, as this measure has proven in previous studies to well represent the socio-economic status [24].

We further used information on health service utilization as secondary endpoint – next to HRQoL -, which was also assessed in the SAPALDIA4 questionnaire. We defined three health care utilization variables: Visit to physician(s); to hospital(s); combination of visits to physician(s) and hospital(s). The information were reported for the 12 months preceding the survey (yes vs. no for each). We treated these response variables as binary because nearly all participants reported 0 or 1 visit in the specified period.

Furthermore, we used information on doctor’s diagnosis reported in the SAPALDIA4 questionnaires for sensitivity analysis. Specifically, we defined a variable capturing the following cardiovascular diseases: Myocardial Infarction, Angina Pectoris, Heart Insufficiency, Claudicatio Intermittens and Stroke.

### Health-related quality of life (HRQoL) measures

Questionnaires at SAPALDIA3 and SAPALDIA4 also included the 36-Item Short-Form Health Survey (SF-36), a widely used HRQoL assessment tool that was validated in large population-based surveys as well as in clinical settings [25, 26]. The construct as well as the criteria validity and reliability were thoroughly tested in the development of this tool and are therefore given [27, 28].

The questionnaire is designed to provide a summary of physical and mental health scores, based on eight domains. The physical component comprises physical functioning (PF), bodily pain (BP), role-physical (RP) and general health perception (GH). The mental component reflects the vitality (VT), social role functioning (SF), role emotional (RE) and mental health perception (MH). Scores for each subscale range from 0 to 100, and higher scores indicate better HRQoL [29]. Three domains of the SF-36 (social role functioning, role-physical & role emotional) showed only very few distinct values in our sample and the proportion of subjects at SAPALDIA4 with the perfect score 100 ranged from 72 to 85%. As a consequence, we did not consider these variables. As these variables represent minor sub-scales of the SF-36, their exclusion still allowed us to assess MCS and PCS as HRQoL outcomes.

### Health examination

The health examination consisted of measurements of weight (SECA877 flat scale; SAPALDIA3&4), height (SECA206, wall-fixed measuring system; SAPALDIA3&4), hip and waist circumference (SECA201 ergonomic measuring tape, SECA, Reinach, Switzerland; SAPALDIA3&4), bio-impedance analysis (BIA) using two different devices (Helios, Forana GmbH, Frankfurt, Germany (SAPALDIA3&4 Tanita MC-780MA, TANITACorporation, Tokyo, Japan; SAPALDIA4), blood pressure and heart rate measurements (Omron MC6 or Omron 705-IT, Anandic Medical Systems AG, Bern Switzerland; SAPALDIA3&4); non-fasting blood glucose (only measured at SAPALDIA3); diagnostics for HbA1c and triglycerides (SAPALDIA4: point-of-care diagnostics from capillary blood: Afinion AS100 Analyzer; ALERE, Wädenswil, Switzerland; SAPALDIA3: analysis of venous blood) [30, 31].

Blood pressure measurements at SAPALDIA3&4 were taken after the participant was seated for at least 10 min. Two measurements were taken, with a break of 3 min between measurements. The blood measurements for glycemia and triglycerides were taken in a non-fasting state. From the anthropometric measurements, body mass index (BMI) (SAPALDIA3&4) and waist to hip ratio (SAPALDIA3 & 4) were derived.

### Cardio-metabolic physiological functioning clustered in latent classes

Five categorical variables at SAPALDIA3&4, which reflect cardio-metabolic physiological functioning were considered for identifying latent classes. At both time points (SAPALDIA3 & 4) variables with evidence-based thresholds or recommendations were categorized accordingly, namely systolic blood pressure [32] (diastolic blood pressure was omitted due to its high correlation with systolic blood pressure), BMI [33], HbA1c [34] and triglycerides [35]. For percentage body fat, sex-specific tertiles were calculated in the absence of a reference for categorization (Table 1).

**Table 1:** Categorization of physiological functioning variables for LCA at SAPALDIA3 & 4

Variables	Categorization for LCA
Systolic blood pressure	≤120–129 (normal), 130–139 (elevated), ≥140 (hypertensive)
BMI	< 18.5 (underweight), 20- < 25 (normal weight), ≥25–30 (overweight), > 30 (obese)
HbA1c (%)	< 5.7 (desirable), 5.7–6.5(borderline), > 6.5 (high)
Triglycerides (mmol/l)	< 1.7 (desirable), 1.7–2.0 (borderline), > 2.0 (high)
Percentage Body fat (%)	Male: < 26 (low), 26–31 (intermediate), > 31 (high) Female:< 36(low), 36–40 (intermediate), > 40 (high)

*BMI* Body Mass Index, HbA1C, Glycated hemoglobin, *MPA* Moderate physical activity, *VPA* Vigorous physical activity

### Metabolic syndrome

The metabolic syndrome at SAPALDIA3&4 was defined based on the Joint Interim Statement (JIS) [36]. The JIS defines the metabolic syndrome if any three of the following values are present: Blood glucose ≥5.6 mmol/L (medication or diagnosis for elevated blood glucose as alternate indicator); waist circumference ≥ 94 cm in men & ≥80 cm in women; systolic blood pressure ≥ 130 or diastolic blood pressure ≥ 85 (antihypertensive medication or diagnosis as alternate indicator); hdl < 1.0 mmol/L in men & < 1.3 mmol/L in women; triglycerides ≥1.7 mmol/L (medication or diagnosis for dyslipidemia as alternate indicator). At SAPALDIA4 we used Hba1C instead of blood glucose (Hba1C ≥5.7%, medication or diagnosis for elevated blood glucose as alternate indicator), as blood glucose was not measured in this follow-up.

### Statistical analysis

In a first step [1] LCA was carried out to empirically classify cardio-metabolic physiological functioning variables, separately for SAPALDIA3 and SAPALDIA4. Subjects were characterized based on their values of the five predictor variables. LCA was used as an explorative tool (unconstrained LCA) without a priori expectation about the number of classes.

In order to detect the appropriate number of classes and maximize model fit, we started with a one-class model and increased the number of latent classes up to six. The final model was selected by examining the Bayesian information criterion (BIC) and the Akaike Information Criterion (AIC) in the first place. These indices have shown to be useful for determining the appropriate number of classes for LCA [37]. We checked whether there was a good discrimination between the final definition of latent classes and the five predictor variables. Two additional model fit indices, the adjusted BIC and the consistent AIC, were considered along with the proportions of the single classes to further support the decision on the final model. We assessed cross-sectional associations of the derived latent classes at SAPALDIA4 with socio-demographic characteristics of the study population by using multinomial logistic regression models with latent class membership as outcome variable and sex, age, educational level and study area as simultaneous predictor variables.

To assess cross-sectional and longitudinal associations of the latent classes with HRQoL domains and to compare them to according MetS-HRQoL associations, we performed quantile regression models with adjustment for individual predictor variables (e.g. sex, age and educational level) as well as for HRQoL at baseline in the case of longitudinal associations. Quantile regression models were chosen to address the problem of left-skewed distribution of HRQoL measures.

In order to assess confounding and effect modification by physical activity in the above associations, we first ran the models with and without adjustment for physical activity and second created composite variables classifying participants based on a combination of latent classes or MetS with physical activity status.

Finally, we used logistic regression models to assess cross-sectional associations of the composite variables with health service utilization.

For sensitivity analyses we reran analyses by excluding subjects with self-reported diagnoses of a cardiovascular disease at SAPALDIA4 (Myocardial Infarction, Angina Pectoris, Heart Insufficiency, Claudicatio Intermittens, Stroke).

We performed all analyses using Stata 15 (Stata Corporation, College Station, Texas).

## Results

### Characteristics of the study population

The descriptive characteristics of the study population can be seen in Table 2. Women and men were equally distributed among the subjects at both time points (SAPALDIA3&4). The mean age at SAPALDIA3 was 60.0 ± 7.8 (range: 47–80 years) and at SAPALDIA4 67.4 ± 7.9 (range: 55–88 years). Nearly two-third of the subjects showed medium education levels, which refers to having completed middle school. The mean score of the overall HRQoL domain (GH) showed descriptive difference by sex and with advancing age. With declining education the score decreased. The HRQoL GH scores mostly decreased in a dose-dependent manner with increasingly poorer physiological functioning variables (blood pressure, glycemia, body fat, BMI and triglycerides). Physical activity displayed higher HRQoL GH scores for the sufficiently active subjects. Physical activity and physiological parameters generally decreased from SAPALDIA3 to SAPALDIA4, and therefore with increase in age, while the prevalence of the metabolic syndrome increased.

**Table 2:** Characteristics of the study population

Variable	SAPALDIA3 (*n* = 902)	SAPALDIA4 (*n* = 1124)	Mean score of overall HRQoL (General Health) at SAPALDIA4
Sex			
Male	469 (52%)	561 (50%)	70.4
Female	433 (48%)	563 (50%)	72.4
Age (Mean, SD)	60.0 ± 7.8	67.4 ± 7.9	71.4
Education			
Low	22 (2%)	59 (5%)	69.4
Middle	541 (60%)	705 (63%)	71.5
High	339 (38%)	360 (32%)	71.5
**Physiological functioning**			
BMI			
Low (< 18.5)	9 (1%)	11 (1%)	73.8
Normal (20- < 25)	412 (46%)	467 (42%)	72.4
Overweight (≥25–30)	352 (39%)	449 (40%)	71.8
Obese (> 30)	129 (14%)	197 (18%)	68.0
Body Fat (%)			
Low	302 (33%)	375 (33%)	73.2
Intermediate	300 (33%)	375 (33%)	72.1
High	300 (33%)	374 (33%)	68.5
Triglycerides			
Normal	548 (61%)	668 (59%)	72.3
Borderline	89 (10%)	127 (11%)	71.8
High	265 (29%)	329 (29%)	69.4
Glycemia (HbA1c)			
Desirable	819 (91%)	775 (69%)	72.2
Borderline	69 (8%)	297 (26%)	70.3
High	14 (33%)	52 (5%)	65.5
Blood pressure (systolic)			
Normal	421 /47%)	446 (40%)	72.4
Elevated	182 (20%)	236 (21%)	70.6
Hypertensive	299 (33%)	442 (39%)	70.9
**Physical Activity Guidelines (WHO)**			
Inactive	187 (21%)	352 (31%)	67.4
Sufficiently active	715 (79%)	772 (69%)	73.3
Prevalence of the MetS	287 (32%)	499 (44%)	

Education: Low = Primary School (≤ 9 years), Middle = Secondary school, middle school or apprenticeship (≤12 years), High = Technical College or University (≥12 years)

Physical Activity Guidelines (WHO)

Inactive: < 150 min of MPA and < 75 VPA per week

Sufficient: > 150 min of MPA or > 75 VPA per week

HbA1c (%):< 5.7 (desirable), 5.7–6.5(borderline), > 6.5 (high)

Triglycerides (mmol/l): < 1.7 (desirable), 1.7–2.0 (borderline), > 2.0 (high)

Percentage Body fat (%): Male: 10–26 (low), 26–31 (intermediate), > 31 (high)

Female: 9–36(low), 36–40 (intermediate), > 40 (high)

Systolic blood pressure: 120–129 (normal), 130–139 (elevated), ≥140 (hypertensive)

MetS = Metabolic Syndrome: Any three of the following values: Blood glucose ≥5.6 mmol/L (Hba1C ≥5.7 in SAPALDIA4); waist circumference ≥ 94 cm in men & ≥80 cm in women; systolic blood pressure ≥ 130 or diastolic blood pressure ≥ 85; hdl < 1.0 mmol/L in men & < 1.3 mmol/L in women; triglycerides ≥1.7 mmol/L

### Model fit statistics for LCA

At both time points (SAPALDIA3 & 4) the main values of the BIC and AIC reached its minimum in the model consisting of three classes. The adjusted BIC as well as the CAIC supported these results (Additional Tables 2 & 3). By considering these goodness-of-fit indices the model with three latent classes was chosen to be the final model. The model with three classes also generated a relatively even distribution at SAPALDIA4 (N_1_ = 340 N_2_ = 455, N_3_ = 329) within the study sample, which increases statistical power, while the SAPALDIA3 sample showed fewer subjects in the third class (N_1_ = 412, N_2_ = 352, N_3_ = 137).

### Three cardio-metabolic physiological functioning clusters defined by LCA

The proportion of the different classes and the estimated class-specific probabilities for the predictor variables are shown in Additional Table 4 (SAPALDIA4) and Additional Table 5 (SAPALDIA3). The by-latent-class distribution of variables included in deriving the classes was comparable between SAPALDIA3 and SAPALDIA4.

Class 1, labeled “Healthy” consisted of individuals having mainly normal physiological functioning measured such as blood pressure, glycemia, triglycerides and percentage body fat or BMI. This class included 30% of participants at SAPALDIA4 and 46% of participants at SAPALDIA3.

In Class 2, labeled “At risk”, most individuals were overweight and had intermediate percentages of body fat. The prevalence of high blood pressure, hyperglycemia, and dyslipidemia in this group were intermediate compared to those observed for classes 1 and 3. Most subjects at SAPALDIA4 were categorized into this class (42%), while at SAPALDIA3 this class consisted of 29% of the participants.

Class 3, labeled “Unhealthy” had an overrepresentation of individuals that were obese, in contrast to the other classes that contained no obese participants. Accordingly, participants in class 3 were more likely to have a high percentage of body fat, to be hypertensive, to have high glycemia and triglyceride values. In the SAPALDIA4 sample 29% was assigned to this class, while in the SAPADALDIA3 sample only 15%.

In regards to the socio-demographic distribution of the latent classes at SAPALDIA4 (Table 3) we observed that females were less likely to belong to the Unhealthy or the At risk class. The likelihood of being categorized as “Healthy” decreased with age and lower educational level. Persons aged 75+ or with low educational level were particularly likely to be categorized as Unhealthy. The descriptive data of sex, age and education between the different classes can be seen in Additional Table 6.

**Table 3:** Association of socio-demographic characteristics with the latent classes (SAPALDIA4)

	Sex		Age (years)			Education		
Latent Classes	Male	Female	55–64	65–75	75+	Low	Middle	High
	RRR (95%CI)							
Healthy (Reference)								
At risk	Ref	0.51* (0.38; 0.69)	Ref	1.19 (0.87; 1.63)	1.59* (1.04; 2.44)	Ref	0.50 (0.38; 1.69)	0.32* (0.14; 0.76)
Unhealthy	Ref	0.47* (0.34; 0.66)	Ref	2.02* (1.43; 2.87)	2.50* (1.58; 3.96)	Ref	0.30* (0.13; 0.69)	0.20* (0.080; 0.48)

RRR = Relative Risk Ratio; * = *p* < 0.05

Relative risk ratios are mutually adjusted for all variables included in the model as well as for study area

### Cross-sectional associations of latent classes and MetS with HRQoL domains, without and with additional adjustment for physical activity (SAPALDIA4)

The results of the quantile regression analyses (Table 4A) showed that, compared with the reference class (Healthy) median HRQoL scores were lower in the Unhealthy class, i.e., with differences of − 3.06 (95%CI: (− 5.51, − 2.14) in the GH domain, of − 5.64 (− 6.98, − 4.30) in the PF domain and of − 9.14 (− 13.91, − 4.38) in the BP domain. The At risk class only had borderline significant scores in the PF domain (− 1.01 (− 2.05; − 0.03)), compared to the reference class. The association results of the latent classes with HRQoL did not substantially differ when adjusting for physical activity in the model, although they tended to become somewhat weaker. We observed statistically significant positive associations for being physically active (i.e. meeting WHO guidelines) with all HRQoL domains. Coefficients ranged from 3.92 to 7.50 for the comparison of meeting vs. not meeting WHO physical activity guidelines. The median scores and descriptive differences of the latent classes (without adjustment of physical activity) can be found in Additional Fig. 1.

**Table 4:** Cross-sectional adjusted associations of latent classes (4A) and metabolic syndrome (4B) with HRQoL domains, without and with additional adjustment for physical activity (SAPALDIA4)

*N* = 1124	General Health (GH)		Physical Functioning (PF)		Bodily Pain (BP)		Vitality (VT)		Mental Health (MH)	
	Coefficient (95% CI)	*P*-value	Coefficient (95% CI)	P-value	Coefficient (95% CI)	P-value	Coefficient (95% CI)	P-value	Coefficient (95% CI)	P-value
4A. Latent classes										
Healthy	Reference									
At risk	−0.22 (−2.57; 2.14)	0.855	−1.01 (− 2.05; − 0.03)	0.057	− 1.39 (− 4.17; 1.40)	0.329	−0.00 (− 2.00; 2.00)	1.000	−0.30 (− 1.97; 1.16)	0.721
Unhealthy	−3.06 (− 5.51; − 0.58)	0.015	− 5.64 (− 6.98; − 4.30)	< 0.001	−9.14 (− 13.91; − 4.38)	< 0.001	− 2.50 (− 5.22; − 0.22)	0.072	− 1.10 (− 3.37; 1.16)	0.339
***+ Physical Activity***										
Healthy	Reference									
At risk	0.12 (− 2.28; 2.52)	0.924	− 0.73 (− 1.65; 0.18)	0.115	−1.43 (−4.26; 1.39)	0.319	− 2.50 (− 4.52; − 0.48)	0.092	0.00 (− 1.93; 1.93)	1.000
Unhealthy	− 2.64 (− 5.29; 0.00)	0.050	− 5.00 (− 6.59; − 3.41)	< 0.001	−7.82 (− 12–48; − 3.16)	0.001	− 2.50 (− 4.76–0.24)	0.030	0.00 (− 2.46; 2.46)	1.000
Physical Activity (Sufficiently active)	4.27 (1.98; 6.56)	< 0.001	3.92 (2.50; 5.34)	< 0.001	4.27 (0.67; 7.87)	0.020	7.50 (5.28; 9.71)	< 0.001	4.00 (1.50; 6.50)	0.002
4B. Metabolic Syndrome (MetS)										
No MetS	Reference									
Yes MetS	−3.47 (−5.41; −1.54)	< 0.001	− 3.46 (−4.69; −2.22)	< 0.001	− 2.69 (−6.39; 1.01)	0.153	− 5.00 (−7.18; − 2.82)	< 0.001	− 0.21 (− 1.99; 1.57)	0.817
***+ Physical Activity***										
Yes MetS	− 3.18 (− 5.27; − 1.08)	0.003	− 2.40 (− 3.73; − 1.08)	< 0.001	− 2.92 (− 6.23; 0.39)	0.084	−2.50 (− 4.12; − 0.88)	0.003	0.00 (−1.76; 1.76)	1.000
Physical Activity (Sufficiently active)	4.11 (1.69; 6.53)	0.001	4.03 (2.60; 5.45)	< 0.001	5.70 (2.88: 8.52)	< 0.001	7.50 (5.32; 9.68)	< 0.001	4.00 (1.44; 6.56)	0.002

CI = Confidence interval

Differences in median levels were analysed with quantile regression models; a negative effect indicates a lower median score

Adjusted for sex, age, education level and study area

Categorized according to WHO Physical Activity Guidelines

Inactive: < 150 min of MPA and < 75 VPA per week

Sufficiently active: > 150 min of MPA or > 75 VPA per week.

Metabolic syndrome: Any three of the following values: Blood glucose ≥5.6 mmol/L (Hba1C ≥5.7% in SAPALDIA4); waist circumference ≥ 94 cm in men & ≥80 cm in women; systolic blood pressure ≥ 130 or diastolic blood pressure ≥ 85; hdl < 1.0 mmol/L in men & < 1.3 mmol/L in women; triglycerides ≥1.7 mmol/L

Participants with MetS showed associations in the same direction with HRQoL domains as the Unhealthy group (Table 4B). Neither MetS nor the class “Unhealthy” were associated with the MH domain. The associations did not differ substantially after adjustment for physical activity. As in the model with the latent classes, physically active subjects had higher HRQoL scores in all domains.

### Cross-sectional HRQoL associations of composite variables combining physical activity with latent classes and MetS, respectively (SAPALDIA4)

**Fig. 2:**
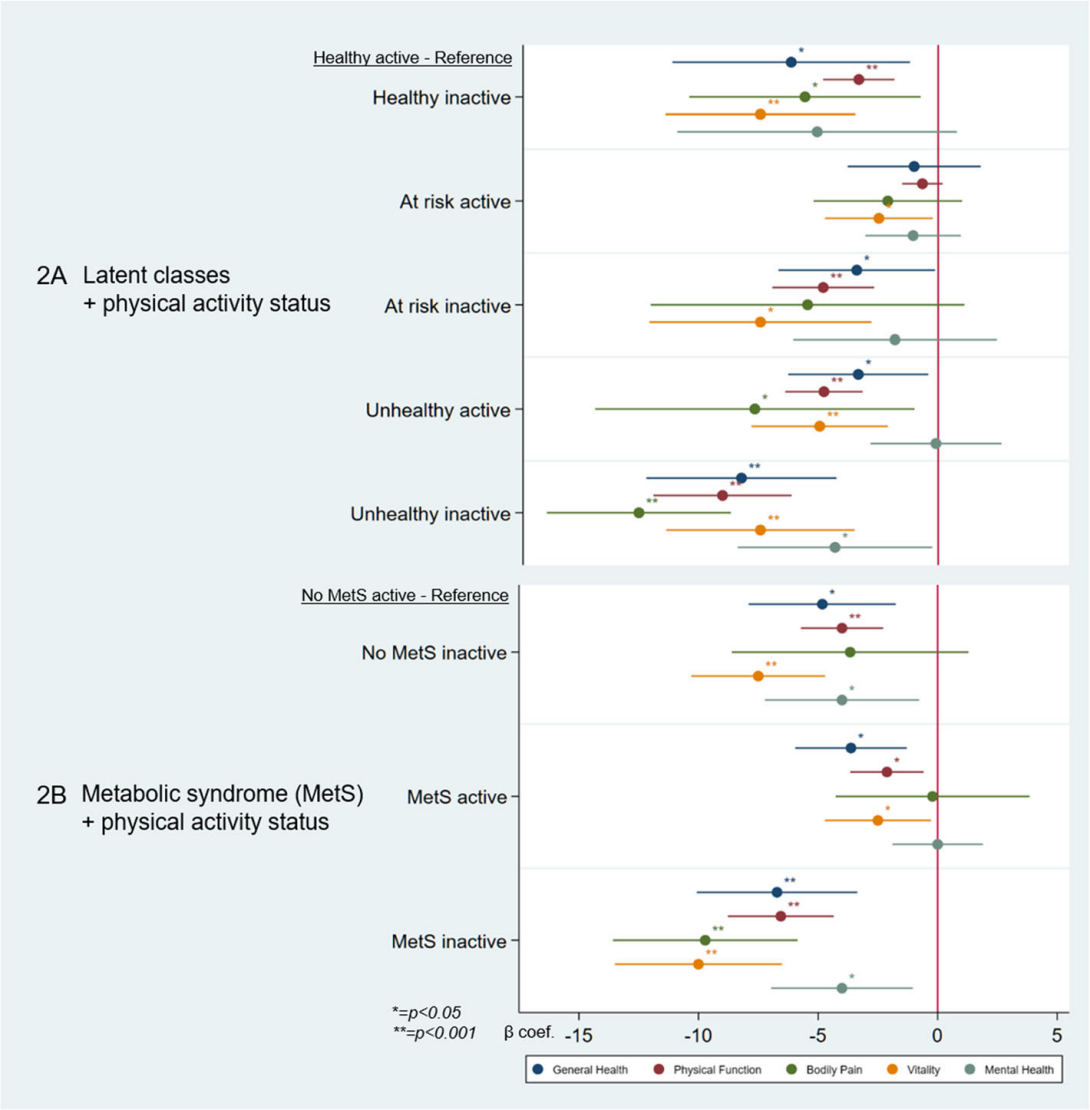
Cross-sectional adjusted HRQoL associations of categories combining physical activity with latent classes (2A) as well as metabolic syndrome (2B) (SAPALDIA4)

Figure 2 shows the results of the quantile regression models of for the composite variables cross-categorizing participants based on their physical activity and their latent class or MetS, respectively, and their association with HRQoL domains. We observed (Fig. 2A) that compared to the reference group (Healthy & active) all categories had coefficients in the direction of lower HRQoL scores, irrespective of the domain considered. Moreover, within latent class categories the inactive groups consistently exhibited lower scores on average than the active one. The lowest HRQoL scores were observed in the unhealthy and physically inactive group compared to the healthy and physically active group for the GH, PF, and BP domain. This group even exhibited statistically significantly lower scores in the MH domain compared to the same reference group.

The association patterns for the composite MetS/physical activity variables with HRQoL (Fig. 2B) were consistent with the association patterns for the composite variable considering latent classes instead of MetS.

The exact coefficients for Fig. 2A and B can be seen in Additional Table 7.

### Prospective associations of composite variables combining physical activity with latent classes and MetS, respectively, at SAPALDIA3 with HRQoL at SAPALDIA4

The longitudinal results are displayed in Table 5. We observed statistically significant differences compared to the Healthy & active group of − 4.95 (95% CI: − 7.01; − 2.90) points in effect score for the association of the Unhealthy & inactive group with GH. Furthermore, the Unhealthy & active group scored − 2.47 (− 4.32; − 0.81) points in effect score less in the PF domain compared to the reference group. The At risk & inactive group showed statistically significantly lower scores for the VT domain of − 2.29 (− 4.34; − 0.23) points in effect scores. Similar to Table 4 we detected generally lower HRQoL scores in inactive participants in the Unhealthy group, but the same pattern was not as consistently observed in the Healthy and At risk groups.

**Table 5:** Prospective associations of composite variable categories defined by latent classes (Table A9A) and metabolic syndrome (Table A9B), respectively, and physical activity status at SAPALDIA3, with median levels of HRQoL at SAPALDIA4, adjusted for respective HRQoL domain at SAPALDIA3

*N* = 902	General Health (GH)		Physical Functioning (PF)		Bodily Pain (BP)		Vitality (VT)		Mental Health (MH)	
	Coefficient (95% CI)	*P*-value	Coefficient (95% CI)	*P*-value	Coefficient (95% CI)	*P*-value	Coefficient (95% CI)	*P*-value	Coefficient (95% CI)	*P*-value
5A. Latent classes + Physical Activity										
Healthy & active	Reference									
Healthy & inactive	−0.36 (−2.56; 2.84)	0.749	− 0.24 (− 1.69; 1.21)	0.741	0.00 (−4.69; 4.69)	1.000	− 0.32 (−3.64; 3.00)	0.849	0.57 (− 3.69; 4.83)	0.792
At risk & active	−2.02 (− 4.50; 0.40)	0.100	− 0.69 (− 1.81; 0.42)	0.223	0.00 (− 1.72; 1.72)	1.000	−1.00 (− 3.11; 1.13)	0.359	−0.57 (− 2.20; 1.06)	0.492
At risk & inactive	−0.92 (− 4.05; 2.21)	0.564	− 1.03 (− 2.52; − 0.46)	0.175	−0.62 (− 4.95; 3.71)	0.778	− 1.29 (− 4.93; 2.34)	0.485	− 2.29 (− 4.34; − 0.23)	0.029
Unhealthy & active	− 1.63 (− 4.03; 0.76)	0.181	−2.57 (− 4.32; − 0.81)	0.004	−0.69 (− 4.71; 3.33)	0.737	− 0.13 (− 2.70; 2.45)	0.922	0.00 (− 2.64; 2.64)	1.000
Unhealthy & inactive	−4.95 (− 7.01; − 2.90)	< 0.001	− 3.55 (− 8.41; 1.31)	0.152	−5.42 (− 15.45; 4.61)	0.289	0.04 (− 3.71; 3.80)	0.981	1.71 (− 3.88; 7.31)	0.547
5B. Metabolic Syndrome + Physical Activity										
No MetS & active	Reference									
No MetS & inactive	1.03 (− 1.28; 3.34)	0.381	0.32 (−0.79; 1.44)	0.569	0.00 (−4.67; 4.67)	1.000	1.64 (−0.83; 4.11)	0.192	0.55 (−2.08; 3.18)	0.663
MetS & active	−0.43 (−2.38; 1.53)	0.668	− 1.79 (− 2.80; − 0.79)	< 0.001	0.00 (− 1.27; 1.27)	1.000	1.74 (− 0.16; 3.64)	0.072	1.92 (0.31: 3.53)	0.020
MetS & inactive	−4.83 (−7.89; − 1.78)	0.002	− 2.48 (− 4.46; − 0.50)	0.014	− 1.38 (−5.42; 2.66)	0.504	− 2.19 (− 5.80; 1.43)	0.235	0.51 (− 2.97; 3.98)	0.776

CI = Confidence interval

Differences in median levels were analysed with quantile regression models; a negative effect indicates a lower median score

Adjusted for sex, age, education level, study area, and HRQoL at SAPALDIA3

Categorized according to WHO Physical Activity Guidelines

Inactive: < 150 min of MPA and < 75 VPA per week

Sufficiently active: > 150 min of MPA or > 75 VPA per week

Metabolic syndrome: Any three of the following values: Blood glucose ≥5.6 mmol/L (Hba1C ≥5.7% in SAPALDIA4); waist circumference ≥ 94 cm in men & ≥80 cm in women; systolic blood pressure ≥ 130 or diastolic blood pressure ≥ 85; hdl < 1.0 mmol/L in men & < 1.3 mmol/L in women; triglycerides ≥1.7 mmol/L

In Table 5B we detected no differences in HRQoL scores by physical activity status among the participants without MetS. In contrast, among persons with MetS, HRQoL scores were consistently lower for inactive participants. The difference reached statistical significance for GH, and PF. In the MetS group the sufficiently active and inactive, respectively, had − 0.43 (95%CI: − 2.38; 1.53) and − 4.83 (− 7.89; − 1.78) lower scores for GH as well as − 1.79 (− 2.80; − 0.79) and − 2.48 (− 4.46; − 0.50) lower scores for PF, when compared to active subjects without MetS. The Mets & active group showed statistically significant differences, compared to the reference group (NoMetS & active) in the MH domain of 1.92 (0.31: 3.53).

### Associations of composite variables combining physical activity with latent classes and MetS, respectively, with health service utilization (SAPALDIA4)

Table 6A displays the association of composite variables with self-reported health service use in the year before SAPALDIA4. While no associations were observed with physician’s visits, participants in the Unhealthy & inactive group (OR 2.69 (1.52; 4.74)) and At risk & inactive (2.09 (1.14; 3.81)) group exhibited the highest risk for a hospitalization in the previous 12 months compared to participants in the reference group (Healthy & active).

**Table 6:** Cross-sectional associations of composite variable categories defined by latent classes (Table A10A) and metabolic syndrome (Table A10B), respectively, and physical activity status with health service utilization in the last 12 months (SAPALDIA4)

*N* = 1124	Physician visit		Hospital visit		Combined	
6A. Latent classes + Physical Activity	Odds ratio (95% CI)	P-value	Odds ratio (95% CI)	P-value	Odds ratio (95% CI)	P-value
Healthy & active	Reference					
Healthy & inactive	1.22 (0.59; 2.53)	0.596	1.79 (0.89; 3.59)	0.101	1.15 (0.55; 2.40)	0.702
At risk & active	1.02 (0.65; 1.60)	0.927	0.97 (0.57; 1.64)	0.910	0.99 (0.63; 1.57)	0.980
At risk & inactive	1.11 (0.61; 2.03)	0.722	2.09 (1.14; 3.81)	0.017	1.05 (0.58; 1.92)	0.868
Unhealthy & active	1.68 (0.96; 2.95)	0.069	1.44 (0.81; 2.53)	0.211	1.64 (0.93; 2.91)	0.089
Unhealthy & inactive	1.03 (0.58; 1.85)	0.908	2.69 (1.52; 4.74)	0.001	1.13 (0.62; 2.06)	0.701
6B. Metabolic syndrome (MetS) + Physical activity						
No MetS & active	Reference					
No MetS & inactive	0.90 (0.55; 1.47)	0.673	2.26 (1.38; 3.72)	0.001	0.85 (0.52; 1.39)	0.527
MetS & active	1.64 (1.07; 2.54)	0.024	1.52 (0.98; 2.38)	0.060	1.54 (0.99; 2.38)	0.053
MetS & inactive	1.49 (0.90; 2.45)	0.120	2.63 (1.64; 4.22)	< 0.001	1.57 (0.93; 2.66)	0.093

CI = Confidence interval

Probabilities were calculated using logistic regression models for binary outcomes

Outcomes: 0 vs. ≥1

Adjustued Variables: Sex, age, education level and study area

Physical Activity Guidelines (WHO)

Inactive: < 150 min of MPA and < 75 VPA per week

Sufficiently active: > 150 min of MPA or > 75 VPA per week.

Metabolic syndrome: Any three of the following values: Blood glucose ≥5.6 mmol/L (Hba1C ≥5.7% in SAPALDIA4); waist circumference ≥ 94 cm in men & ≥80 cm in women; systolic blood pressure ≥ 130 or diastolic blood pressure ≥ 85; hdl < 1.0 mmol/L in men & < 1.3 mmol/L in women; triglycerides ≥1.7 mmol/L

The risk of hospitalization was also strongest for the group MetS & inactive (2.63 (1.64; 4.22)) and No MetS & inactive group (2.26 (1.38; 3.72)), compared to the reference group (Table 6B). Same as for the latent classes, the associations with physician’s visit did not show a pattern of a higher likelihood among inactive persons without MetS.

### Sensitivity analyses

In the sub-sample of subjects not reporting any cardiovascular diseases we found cross-sectional and longitudinal associations with HRQoL and cross-sectional associations with health service utilization comparable to those in the overall sample for both, composite variables based on latent classes and on MetS, respectively (Additional Table 8 and 9 for HRQoL; Additional Table 10 for health service utilization).

## Discussion

In this general population sample from Switzerland, HRQoL derived from SF-36 was generally high compared to similar settings and age groups [38].

The use of LCA enabled us to cluster physiological functioning of the SAPALDIA general population sample and identify three latent classes that were comparable over two time points almost 10 years apart (SAPALDIA3&4). This longer-term clustering of physiological functioning is likely the result of temporal tracking of lifestyle and behavioral habits. The class termed “Unhealthy” characterized with a high percentage of participants with obesity and a high percentage of body fat in the presence of cardio-metabolic pathophysiology was associated with considerably lower HRQoL scores in all physical component scores of HRQoL. The largest difference to the “Healthy” reference class in terms of median scores was found for the PF and BP domains, which were also lower in the “At risk” class. Differences in HRQoL between classes were larger for physical compared to mental health components. Yet, the VT subdomain, belonging to the mental health component also showed considerable differences for the Unhealthy class compared to the Healthy class. Even though the coefficients were smaller and in part statistically not significant in the longitudinal analysis, we found similar results as in the cross-sectional analyses. These consistent results give an indication on prospective pathways leading from the latent classes to decreased HRQoL. As we adjusted for HRQoL at baseline (SAPALDIA3), we may also argue that reverse causality has been partly excluded, even though much more evidence is needed to prove this hypothesis. Of interest from a primary prevention perspective is the fact that the observed cross-sectional and longitudinal associations were not driven by the sub-group of persons who had already progressed to a clinical diagnosis of cardiovascular disease. Our results thus underline the relevance and also opportunity to improve HRQoL at older age through interventions that promote a healthy lifestyle in the “middle-aged” group.

The risk factors being more prevalent in members of the “Unhealthy Class” agreed well with the cluster of risk factors defining the MetS [13]. Although there is not yet much, and mostly cross-sectional evidence on an association of MetS with HRQoL, most studies showed a worsening effect of the MetS on HRQoL, which we support with our findings. However, it was also observed that the MetS alone without accompanying comorbidities such as depression had little or no effect on HRQoL [39–41]. In our study, we observed that the MetS was associated with lower HRQoL and also a higher rate of hospitalization in the 12 month period before the assessment, even in the absence of cardiovascular disease. Our results are in line with recent intervention studies demonstrating that lifestyle changes can improve HRQoL among persons with MetS [14, 42]. Additional longitudinal research is needed to elaborate more on possible causal pathways.

In this study, we were specifically interested in assessing whether the promotion of physical activity in aging populations can attenuate the short- and longer-term adverse consequences of poor physiological functioning and of the MetS on HRQoL. As previously reported, physical activity levels were high in this population (73% of participants meeting WHO guidelines [43]), but aligned with the results of the Swiss Health Survey, which reported 76% of adults being sufficiently active in Switzerland [44]. Consistent with the literature, we observed strong and independent positive associations of physical activity with HRQoL [17, 21, 45, 46].

We observed in a remarkably consistent way that physical inactivity was associated with lower HRQoL scores in many domains, both in all latent classes and in persons with and without MetS. For the physical functioning domains, HRQoL scores were mostly lowest in physically inactive persons assigned to the Unhealthy class as well as combined with the presence of the MetS. These results are in line with the few randomized controlled trials demonstrating the benefit of physical activity for HRQoL in subjects with MetS or cardiovascular diseases [47–50].

The direct associations of physical activity with HRQoL and the moderating influence of physical activity on the association of the “Unhealthy” latent class as well as of MetS with HRQoL underline the pivotal role of physical activity in preventing and mitigating diseases [51]. Physical activity is of crucial importance, not only from a medical perspective, but also from a health-economic perspective, as it represents the most cost-effective approach in the prevention and rehabilitation of cardio-metabolic and aging-induced comorbidities [42, 51, 52]. In this study we found in fact the highest risk for a recent hospitalization in persons exhibiting both, MetS or “Unhealthy” latent class as well as physical inactivity, even in the absence of a cardiovascular diagnosis.

Results from previous research on the association of single components making up the “Unhealthy” class with HRQoL domains are mostly consistent with our findings [11, 12]. Even after controlling for other important health-risk behaviors, an increased BMI seems to be most strongly associated with adverse HRQoL outcomes [6, 17]. A recent systematic review supports these findings and stated that obesity is associated with lower HRQoL outcomes [53]. Another systematic review showed that being overweight or obese resulted in fewer points in the main domains of HRQoL [4], in line with our results. Next to this, hyperglycemia was shown to have direct and indirect effects through inadequate glycemic control on HRQoL outcomes in diabetic elderly [54]. Intervention plans to promote lifestyle changes prevented frailty in diabetic individuals and enhanced life expectancy and HRQoL [55].

In this study, we observed a clear socio-economic gradient across the three latent classes, with participants at the lowest educational level being over-represented in the class with the lowest HRQoL scores. Socio-economic gradients have previously been identified for obesity and associated lifestyles [56–62], for the MetS [63–65], and for HRQoL [66, 67]. Factors that are likely to contribute to these social gradients include the understanding of healthy habits, access to healthy habits (e.g. access to fitness centers; prices for healthy vs. unhealthy food; green spaces and built environment) and access to health services (i.e. regular testing of blood pressure, glycemia, lipids) as well as access to adequate control of disease (i.e. blood pressure medication) [56–58].

### Strength and limitations

The information on socio-economic status and physical activity were self-reported by the participants, which could lead to measurement errors. The fact that participants at SAPALDIA3 were interviewed, whereas at SAPALDIA4 participants self-administered questionnaires, could have introduced differential misclassification of physical activity. But it is unlikely that this would have altered the main messages of our study, given the consistency of longitudinal and cross-sectional results. All other variables included in the analysis were objectively assessed. Triglyceride and glycemia tests and the type of blood as test source (venous versus capillary blood) differed between SAPALDIA3 and SAPALDIA4. While this could again be the source of differential misclassification over time, the consistency of the cross-sectional and longitudinal results suggests this bias to be minimal. The variables reflecting cardio-metabolic physiological functioning were chosen based on the evidence reported in the literature. Our current LCA may furthermore be sensitive to the chosen categories for the predictor variables. Yet, the consistency in results between latent classes and MetS points to the fact that the chosen cutoffs for categorization are clinically relevant. As in any cohort, we cannot exclude bias due to loss-to-follow-up. Several of the factors leading to or included in the categorization of participant’s physiological functioning influenced participation at follow-up. Persons with healthy habits were more likely to participate in SAPALDIA4. Older persons were less likely to participate at follow-up, in part because they had died. It is therefore likely that the effect of poor health habits including obesity and physiological functioning on HRQoL was underestimated. Given this loss-to-follow-up, the SAPALDIA4 participants are most likely no longer representative of the current general population in Switzerland. Yet, with regard to physical activity the observed distribution in our study sample is highly comparable to the distribution recently observed in the population-based Swiss Health Survey. The generalizability of our findings to other study settings needs testing, particularly in the light of the very high physical activity level of the Swiss population in general [43].

Despite these limitations, the findings of this study strongly support that it is possible to reduce the number of physiological functioning variables using LCA. The latent classes corresponded well with the cluster of the MetS, which underlines the significant interplay of these variables.

The LCA was facilitated by access to data from the SAPALDIA participants, who are deeply characterized on various physiological domains. The population-based design of the study is an important prerequisite for the generalizability of the findings, at least within the Swiss setting, although participation and survivor bias always pose a threat to validity and generalizability of any long-running cohort. Finally, the longitudinal design of this study adds knowledge to possible pathways in the investigated associations.

## Conclusion

This study, investigating associations of HRQoL with cardio-metabolic physiological functioning parameters combined with physical activity status in a general population sample, emphasizes the relevance of promoting primary and secondary prevention to maintain well-being in aging populations. In a country like Switzerland with sufficient economic resources, the lack of investments into primary and secondary prevention [68] seems to come at the cost of poor well-being in persons in the second half of their life. This may lead to high treatment costs later in life and to loss of productivity in persons still actively engaged in the workforce [69, 70].

Furthermore, the underrepresentation of primary prevention and screening programs in disease control leads to the clustering of factors among the lower social classes and therefore to inequality in HRQoL despite the strong economy of Switzerland. In the light of increased promotion of personalized medicine as opposed to public health and prevention programs, as well as imbalanced investments into medical treatments, our results may help to understand and guide how to avoid further widening of medical inequalities. This study primarily underlines the importance of physical activity promotion on health and quality of life outcomes of the factors making up the metabolic syndrome.

## Supplementary information

**Additional file 1: Additional Table 1.** Baseline characteristics at SAPALDIA 1 of participants who reached the age of 55+ at the time of the SAPALDIA4 55+ health assessments, stratified by participation status in the current study. **Additional Table 2.** Summary of model fit indices for 1 to 6 latent classes at SAPALDIA4. **Additional Table 3.** Summary of model fit indices for 1 to 6 latent classes at SAPALDIA3. **Additional Table 4.** Proportions and class-specific probabilities for the 3 latent classes at SAPALDIA4. **Additional Table 5.** Proportions and class-specific probabilities for the 3 latent classes at SAPALDIA3. **Additional Table 6.** Descriptive differences in sex, age and education between the three latent classes, SAPALDIA4. **Additional Table 6.** Descriptive differences in sex, age and education between the three latent classes, SAPALDIA4. **Additional Fig. 1.** Descriptive differences of median HRQoL scores of the three latent classes (without adjustment for physical activity) at SAPALDIA4. **Additional Table 7.** Cross-sectional adjusted HQRoL associations of categories combining physical activity with latent classes (A7A) as well as metabolic syndrome (A7B) (SAPALDIA4). **Additional Table 8.** Cross-sectional associations of composite variable categories defined by latent classes (A8A) and metabolic syndrome (A8B), respectively, and physical activity status, with median levels of HRQoL, subjects not reporting any cardiovascular disease (SAPALDIA4). **Additional Table 9.** Prospective associations of composite variable categories defined by latent classes (A9A) and metabolic syndrome (A9B), respectively, and physical activity status at SAPALDIA3, with median levels of HRQoL at SAPALDIA4, adjusted for respective HRQoL domain at SAPALDIA3, subjects not reporting any cardiovascular at SAPALDIA4. **Additional Table 10.** Cross-sectional associations of composite variable categories defined by latent classes (A10A) and metabolic syndrome (A10B), respectively, and physical activity status with health service utilization in the last 12 months, subjects not reporting any cardiovascular (SAPALDIA4).

## Data Availability

Not applicable.

## Abbreviations

HRQoL: Health-related quality of life
LCA: Latent class analysis
MetS: Metabolic syndrome
BMI: Body mass index
SF-36: Short-Form Health Survey
QoL: Quality of life
SAPALDIA: Swiss Study on Air Pollution and Lung and Heart Diseases
PF: Physical functioning
BP: Bodily pain
RP: Role-physical
GH: General health
VT: Vitality
SF: Social role functioning
RE: role emotional
MH: Mental health
BIA: Bio-impedance analysis
BIC: Bayesian information criterion
AIC: Akaike Information Criterion
CI: Confidence interval
