# Supplementary material for: The modifying role of physical activity in the cross-sectional and longitudinal association of health-related quality of life with physiological functioning-based latent classes and metabolic syndrome
Source: Health Qual Life Outcomes. 2020 Oct 20;18:345. doi: 10.1186/s12955-020-01557-z (PMC7574351; doi:10.1186/s12955-020-01557-z)
Supplement: Supplementary file 1 — Additional file 1: Additional Table 1. Baseline characteristics at SAPALDIA 1 of participants who reached the age of 55+ at the time of the SAPALDIA4 55+ health assessments, stratified by participation status in the current study. Additional Table 2. Summary of model fit indices for 1 to 6 latent classes at SAPALDIA4. Additional Table 3. Summary of model fit indices for 1 to 6 latent classes at SAPALDIA3. Additional Table 4. Proportions and class-specific probabilities for the 3 latent classes at SAPALDIA4. Additional Table 5. Proportions and class-specific probabilities for the 3 latent classes at SAPALDIA3. Additional Table 6. Descriptive differences in sex, age and education between the three latent classes, SAPALDIA4. Additional Table 6. Descriptive differences in sex, age and education between the three latent classes, SAPALDIA4. Additional Fig. 1. Descriptive differences of median HRQoL scores of the three latent classes (without adjustment for physical activity) at SAPALDIA4. Additional Table 7. Cross-sectional adjusted HQRoL associations of categories combining physical activity with latent classes (A7A) as well as metabolic syndrome (A7B) (SAPALDIA4). Additional Table 8. Cross-sectional associations of composite variable categories defined by latent classes (A8A) and metabolic syndrome (A8B), respectively, and physical activity status, with median levels of HRQoL, subjects not reporting any cardiovascular disease (SAPALDIA4). Additional Table 9. Prospective associations of composite variable categories defined by latent classes (A9A) and metabolic syndrome (A9B), respectively, and physical activity status at SAPALDIA3, with median levels of HRQoL at SAPALDIA4, adjusted for respective HRQoL domain at SAPALDIA3, subjects not reporting any cardiovascular at SAPALDIA4. Additional Table 10. Cross-sectional associations of composite variable categories defined by latent classes (A10A) and metabolic syndrome (A10B), respectively, and physical activity status with [file 12955_2020_1557_MOESM1_ESM.docx]

| Additional Table 1. Baseline characteristics at SAPALDIA 1 of participants who reached the age of 55+ at the time of the SAPALDIA4 55+ health assessments, stratified by participation status in the current study. | | | | | | | |
| --- | --- | --- | --- | --- | --- | --- | --- |
|  | Non-participation in 55+ health assessment | |  | Participation in 55+ health assessment | |  |  |
|  | N | % |  | N | % |  | p-value |
| **Sex** |  |  |  |  |  |  |  |
| male | 3266 | 48.0 |  | 584 | 50.0 |  | 0.187 |
| female | 3545 | 52.1 |  | 583 | 50.0 |  |  |
| **Language** |  |  |  |  |  |  |  |
| German | 3607 | 53.0 |  | 597 | 51.2 |  | <0.001 |
| French | 2224 | 32.7 |  | 468 | 40.1 |  |  |
| Italian | 980 | 14.4 |  | 102 | 8.7 |  |  |
| **BMI [kg/m^2^}** |  |  |  |  |  |  |  |
| <18.5 | 196 | 2.9 |  | 54 | 4.6 |  | <0.001 |
| <25.0 | 3842 | 57.1 |  | 828 | 71.2 |  |  |
| 25-30 | 2090 | 31.7 |  | 246 | 21.2 |  |  |
| >30 | 599 | 8.9 |  | 35 | 3.0 |  |  |
| **Age (years)** |  |  |  |  |  |  |  |
| 30-40 | 1858 | 27.3 |  | 490 | 42.0 |  | <0.001 |
| 40-50 | 2338 | 34.3 |  | 440 | 37.7 |  |  |
| 50-60 | 2333 | 34.3 |  | 178 | 15.3 |  |  |
| **Education Level** |  |  |  |  |  |  |  |
| low | 1371 | 20.2 |  | 99 | 8.5 |  | <0.001 |
| middle | 4335 | 63.8 |  | 766 | 65.8 |  |  |
| high | 1086 | 16.0 |  | 300 | 25.8 |  |  |
| **Smoking Status** |  |  |  |  |  |  |  |
| never | 2708 | 39.8 |  | 568 | 48.8 |  | <0.001 |
| former | 1736 | 25.5 |  | 317 | 27.2 |  |  |
| current | 2361 | 34.7 |  | 280 | 24.0 |  |  |

Additional Table 2. Summary of model fit indices for 1 to 6 latent classes at SAPALDIA4

| Number of latent classes | BIC | Adjusted BIC | AIC | CAIC | Entropy |
| --- | --- | --- | --- | --- | --- |
| 1 | 1136.9532 | 1102.0142 | 1081.682 | 1136.9532 | 1 |
| 2 | 521.50136 | 448.44702 | 405.93444 | 544.50136 | 0.74059706 |
| 3 | 467.17904 | 356.00937 | 291.31632 | 502.17904 | 0.69212395 |
| 4 | 530.24721 | 380.96224 | 294.08871 | 577.24721 | 0.69434165 |
| 5 | 595.02278 | 407.62249 | 298.56848 | 654.02278 | 0. 65047906 |
| 6 | 655.49728 | 429.98168 | 298.7472 | 726.49728 | 0.7141521 |
| BIC= Bayesian Information Criterion; AIC= Akaike Information Criterion; CAIC= Consistent AIC | | | | | |

Additional Table 3. Summary of model fit indices for 1 to 6 latent classes at SAPALDIA3

| Number of latent classes | BIC | Adjusted BIC | AIC | CAIC | Entropy |
| --- | --- | --- | --- | --- | --- |
| 1 | 963.32393 | 928.3897 | 910.47317 | 974.32393 | 1 |
| 2 | 449.76717 | 376.72288 | 339.26104 | 472.76717 | 0. 76767413 |
| 3 | 418.91023 | 307.75586 | 250.74872 | 453.91023 | 0. 8896 |
| 4 | 488.40881 | 339.14438 | 262.59193 | 535.40881 | 0. 79892745 |
| 5 | 553.71541 | 366.34091 | 270.24316 | 612.71541 | 0. 86553785 |
| 6 | 614.62767 | 389.1431 | 86553785 | 685.62767 | 0. 86315914 |
| BIC= Bayesian Information Criterion; AIC= Akaike Information Criterion; CAIC= Consistent AIC | | | | | |

Additional Table 4. Proportions and class-specific probabilities for the 3 latent classes at SAPALDIA4

| Variables | Healthy  n=340 (30%) | At risk  n= 455 (41%) | Unhealthy  n=329 (29%) |
| --- | --- | --- | --- |
| **BMI** |  |  |  |
| Low | 0.03 | 0.00 | 0.00 |
| Normal | 0.97 | 0.30 | 0.00 |
| Overweight | 0.00 | 0.70 | 0.40 |
| Obese | 0.00 | 0.00 | 0.60 |
| **Body Fat** |  |  |  |
| Low | 0.91 | 0.18 | 0.02 |
| Intermediate | 0.09 | 0.71 | 0.07 |
| High | 0.00 | 0.11 | 0.91 |
| **Triglycerides** |  |  |  |
| Normal | 0.76 | 0.59 | 0.43 |
| Borderline | 0.09 | 0.11 | 0.15 |
| High | 0.15 | 0.31 | 0.42 |
| **Glycemia (HbA1c)** |  |  |  |
| Desirable | 0.83 | 0.73 | 0.49 |
| Borderline | 0.16 | 0.25 | 0.39 |
| High | 0.01 | 0.03 | 0.11 |
| **Blood pressure** |  |  |  |
| Normal | 0.63 | 0.35 | 0.21 |
| Elevated | 0.14 | 0.27 | 0.20 |
| Hypertensive | 0.23 | 0.37 | 0.60 |
| Values represent class-specific probabilities for each variable;  BMI= Body Mass Index; Hba1C= Glycated hemoglobin  HbA1c (%): <5.7 (desirable), 5.7-6.5(borderline), >6.5 (high)  Triglycerides (mmol/l): <1.7 (desirable), 1.7-2.0 (borderline), >2.0 (high)  Percentage Body fat (%): Male: 10-26 (low) , 26-31 (intermediate), >31 (high)  Female: 9-36(low), 36-40 (intermediate), >40 (high)  Systolic blood pressure: 120 -129 (normal), 130 – 139 (elevated), ≥140 (hypertensive) | | | |

Additional Table 5. Proportions and class-specific probabilities for the 3 latent classes at SAPALDIA3

| Variables | Healthy  n=413 (46%) | At risk  n= 352 (29%) | Unhealthy  n=137 (15%) |
| --- | --- | --- | --- |
| **BMI** |  |  |  |
| Low | 0.02 | 0.00 | 0.00 |
| Normal | 0.98 | 0.00 | 0.06 |
| Overweight | 0.00 | 0.100 | 0.00 |
| Obese | 0.00 | 0.00 | 0.94 |
| **Body Fat** |  |  |  |
| Low | 0.61 | 0.13 | 0.01 |
| Intermediate | 0.34 | 0.42 | 0.08 |
| High | 0.05 | 0.44 | 0.91 |
| **Triglycerides** |  |  |  |
| Normal | 0.79 | 0.53 | 0.26 |
| Borderline | 0.06 | 0.11 | 0.18 |
| High | 0.15 | 0.36 | 0.55 |
| **Glycemia (HbA1c)** |  |  |  |
| Desirable | 0.96 | 0.89 | 0.77 |
| Borderline | 0.03 | 0.10 | 0.15 |
| High | 0.01 | 0.01 | 0.07 |
| **Blood pressure** |  |  |  |
| Normal | 0.61 | 0.38 | 0.26 |
| Elevated | 0.17 | 0.20 | 0.29 |
| Hypertensive | 0.22 | 0.41 | 0.44 |
| Values represent class-specific probabilities for each variable;  BMI= Body Mass Index; Hba1C= Glycated hemoglobin  HbA1c (%):<5.7 (desirable), 5.7-6.5(borderline), >6.5 (high)  Triglycerides (mmol/l): <1.7 (desirable), 1.7-2.0 (borderline), >2.0 (high)  Percentage Body fat (%): Male: 10-26 (low) , 26-31 (intermediate), >31 (high)  Female: 9-36(low), 36-40 (intermediate), >40 (high)  Systolic blood pressure: 120 -129 (normal), 130 – 139 (elevated), ≥140 (hypertensive) | | | |

Additional Table 6. Descriptive differences in sex, age and education between the three latent classes, SAPALDIA4

|  | Sex | | Age (years) | | |  | Education | |  |
| --- | --- | --- | --- | --- | --- | --- | --- | --- | --- |
| Latent  Classes | Male | Female | 55-64 | 65-75 | 75+ | | Low | Middle | High |
| Healthy | 140 (25%) | 212 (37%) | 175 (37%) | 131 (28%) | 46 (23%) | | 8 (13%) | 214 (30%) | 130 (36%) |
| At risk | 263 (46%) | 216 (38%) | 202 (42%) | 283 (39%) | 94 (42%) | | 26 (42%) | 309 (43%) | 144 (39%) |
| Unhealthy | 167 (29%) | 146 (25%) | 99 (21%) | 150 (32%) | 64 (31%) | | 28 (45%) | 193 (27%) | 92 (25%) |
| Percentages are displayed within column | | | | | | | | | |


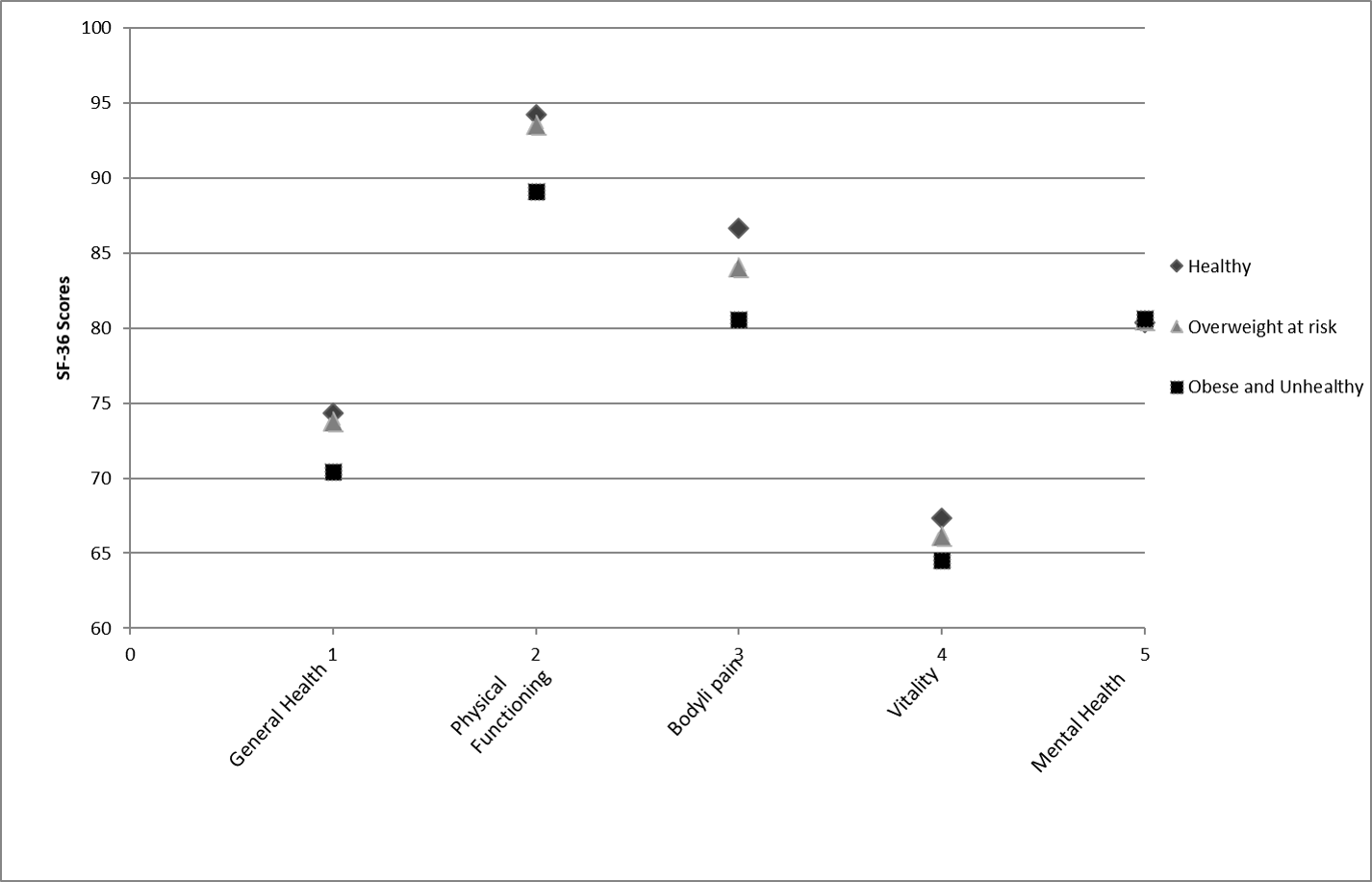


Additional Figure 1. Descriptive differences of median HRQoL scores of the three latent classes (without adjustment for physical activity) at SAPALDIA4

Additional Table 7. Cross-sectional adjusted HQRoL associations of categories combining physical activity with latent classes (A7A) as well as metabolic syndrome (A7B) (SAPALDIA4)

| N=1124 | General Health (GH) | | Physical  Functioning (PF) | | Bodily Pain (BP) | | Vitality (VT) | | | Mental Health (MH) | | | |  |
| --- | --- | --- | --- | --- | --- | --- | --- | --- | --- | --- | --- | --- | --- | --- |
| A7A. Latent classes  + Physical Activity | Coefficient  (95% CI) | P-value | Coefficient (95% CI) | P-value | Coefficient  (95% CI) | P-value | Coefficient  (95% CI) | P-value | | Coefficient  (95% CI) | | P-value | |  |
| Healthy & active | Reference |  |  |  |  |  |  |  | |  | |  | |  |
| Healthy & inactive | -6.20 (-11.22; -1.19) | 0.015 | -3.35 (-4.85; -1.84) | <0.001 | -5.62 (-10.50; -0.74) | 0.024 | -7.50 (-11.51; -3.49) | <0.001 | | -5.11 (-11.00; 0.79) | | 0.090 | |  |
| At risk & active | -1.01 (-3.82; 1.80) | 0.480 | -0.66 (-1.52; 0.19) | 0.129 | -2.12 (-5.26; 1.01) | 0.284 | -2.50 (-4.78; -0.22) | 0.032 | | -1.05 (-3.97; 0.96) | | 0.305 | |  |
| At risk & inactive | -3.43 (-6.74; -0.13) | 0.042 | -4.85 (-7.00; -2.70) | <0.001 | -5.51 (-12.13; 1.11) | 0.103 | -7.50 (-12.18; -2.82) | 0.002 | | -1.82 (-6.12; 2.48) | | 0.406 | |  |
| Unhealthy & active | -3.37 (-6.32; -0.41) | 0.026 | -4.82 (-6.45; -3.19) | <0.001 | -7.74 (-15.48; -0.99) | 0.025 | -5.00 (-7.88; -2.12) | 0.001 | | -0.09 (-2.86; 2.67) | | 0.948 | |  |
| Unhealthy & inactive | -8.30 (-12.31; -4.29) | <0.001 | -9.10 (-12.02; -6.18) | <0.001 | -12.63 (-16.51; -8.74) | <0.001 | -7.50 (-11.48; -3.52) | <0.001 | | -4.35 (-8.46; -0.24) | | 0.038 | |  |
| A7B. Metabolic Syndrome  + Physical Activity | | | | | | | | | | | | | |  |
| No MetS & active | Reference |  |  |  |  |  |  | |  | |  | |  | |
| No MetS & inactive | -4.82 (-7.90; -1.75) | 0.002 | -3.99 (-5.72; -2.28) | <0.001 | -3.66 (-8.61; 1.29) | 0.147 | -7.50 (-10.30; -4.70) | | <0.001 | | -4.00 (-7.22; 0.78) | | 0.015 | |
| MetS & active | -3.62 (-5.96; -1.29) | 0.002 | -2.12 (-3.66; -0.59) | 0.007 | -0.22 (-4.27; 3.84) | 0.917 | -2.50 (-4.72; -0.28) | | 0.027 | | 0.00 (-1.88; 1.88) | | 1.000 | |
| MetS & inactive | -6.72 (-10.07; -3.36) | <0.001 | -6.56 (-8.77; -4.34) | <0.001 | -9.72 (-13.58; -5.86) | <0.001 | -10.00 (-13.49; -6.51) | | <0.001 | | -4.00 (-6.96; -1.04) | | 0.008 | |
| CI= Confidence interval;  Differences in median levels were analysed with quantile regression models; a coefficient of for example of -3.00 means that this group reached -3.00 less points in effect scores in the respective HRQoL domain, compared to the median of the reference group.  Adjustued for sex, age, education level and study area  Categorized according to WHO Physical Activity Guidelines:  Inactive: <150 min of MPA and <75 VPA per week  Sufficiently active: >150 min of MPA or >75 VPA per week  Metabolic syndrome: Any three of the following values: Blood glucose ≥5.6mmol/L (Hba1C ≥5.7% in SAPALDIA4); waist circumference ≥ 94cm in men & ≥80cm in women; systolic blood pressure ≥130 or diastolic blood pressure ≥85; hdl <1.0mmol/L in men & <1.3mmol/L in women; triglycerides ≥1.7 mmol/L. | | | | | | | | | | | | | | |

Additional Table 8. Cross-sectional associations of composite variable categories defined by latent classes (A8A) and metabolic syndrome (A8B), respectively, and physical activity status, with median levels of HRQoL, subjects not reporting any cardiovascular disease (SAPALDIA4)

| N=**921** | General Health (GH) | | Physical  Functioning (PF) | | Bodily Pain (BP) | | Vitality (VT) | | | Mental Health (MH) | | | |  |
| --- | --- | --- | --- | --- | --- | --- | --- | --- | --- | --- | --- | --- | --- | --- |
| A8A. Latent classes  + Physical Activity | Coefficient  (95% CI) | P-value | Coefficient (95% CI) | P-value | Coefficient  (95% CI) | P-value | Coefficient  (95% CI) | P-value | | Coefficient  (95% CI) | | P-value | |  |
| Healthy & active | Reference |  |  |  |  |  |  |  | |  | |  | |  |
| Healthy & inactive | -5.37 (-8.90; -1.84) | 0.003 | -3.19 (-5.03; -1.55) | <0.001 | -7.91 (-11.66; -4.16) | <0.001 | -7.32 (-9.40; -5.24) | <0.001 | | -6.95 (-11.83; -2.07) | | 0.005 | |  |
| At risk & active | -0.98 (-4.12; 2.16) | 0.541 | -0.77 (-1.94; 0.40) | 0.196 | -0.44 (-3.62; 2.74) | 0.786 | -0.91 (-2.95; 1.14) | 0.385 | | 0.25 (-1.77; 2.27) | | 0.808 | |  |
| At risk & inactive | -4.44 (-7.93; -0.95) | 0.013 | -4.22 (-6.40; -2.03) | <0.001 | -6.13 (-13.61; 1.34) | 0.108 | -6.85 (-10.16; -3.54) | <0.001 | | -1.69 (-5.53; 2.14) | | 0.386 | |  |
| Unhealthy & active | -4.27 (-7.75; -0.78) | 0.016 | -4.99 (-6.50; -3.48) | <0.001 | -6.65 (-13.75; 0.46) | 0.067 | -3.84 (-6.01; -1.66) | 0.001 | | -0.14 (-2.91; 2.64) | | 0.923 | |  |
| Unhealthy & inactive | -6.97 (-11.52; -2.41) | 0.003 | -9.29 (-11.96; -6.63) | <0.001 | -9.93 (-13.78; -6.07) | <0.001 | -6.91 (-10.47; -3.35) | <0.001 | | -4.84 (-9.49; -0.10) | | 0.041 | |  |
| A8B. Metabolic Syndrome  + Physical Activity | | | | | | | | | | | | | |  |
| No MetS & active | Reference |  |  |  |  |  |  | |  | |  | |  | |
| No MetS & inactive | -5.15 (-7.87; 2.43) | <0.001 | -3.85 (-5.72; -1.99) | <0.001 | -5.27 (-12.87; 2.34) | 0.147 | -5.00 (-7.85; -2.15) | | 0.001 | | -3.17 (-6.78; 0.44) | | 0.086 | |
| MetS & active | -3.09 (-5.79; -0.39) | 0.025 | -1.63 (-3.09; -0.18) | 0.028 | 0.20 (-3.61; 4.01) | 0.918 | -2.50 (-4.97; -0.03) | | 0.048 | | 0.36 (-1.68; 2.39) | | 0.730 | |
| MetS & inactive | -5.94 (-9.28; -2.61) | <0.001 | -4.79 (-7.11; 2.48) | <0.001 | -10.67 (-12.88; -8.46) | <0.001 | -7.50 (-10-58; -4.42) | | <0.001 | | -3.18 (-6.65; 0.19) | | 0.073 | |
| CI= Confidence interval;  Differences in median levels were analysed with quantile regression models; a coefficient of for example of -3.00 means that this group reached -3.00 less points in effect scores in the respective HRQoL domain, compared to the median of the reference group.  Adjustued for sex, age, education level and study area  Categorized according to WHO Physical Activity Guidelines:  Inactive: <150 min of MPA and <75 VPA per week  Sufficiently active: >150 min of MPA or >75 VPA per week  Metabolic syndrome: Any three of the following values: Blood glucose ≥5.6mmol/L (Hba1C ≥5.7% in SAPALDIA4); waist circumference ≥ 94cm in men & ≥80cm in women; systolic blood pressure ≥130 or diastolic blood pressure ≥85; hdl <1.0mmol/L in men & <1.3mmol/L in women; triglycerides ≥1.7 mmol/L.  Cardiovascular diseases: Myocardial infarction, angina pectoris, heart insufficiency, claudicatio intermittens, stroke | | | | | | | | | | | | | | |

Additional Table 9. Prospective associations of composite variable categories defined by latent classes (A9A) and metabolic syndrome (A9B), respectively, and physical activity status at SAPALDIA3, with median levels of HRQoL at SAPALDIA4, adjusted for respective HRQoL domain at SAPALDIA3, subjects not reporting any cardiovascular at SAPALDIA4

| N=**743** | General Health (GH) | | Physical  Functioning (PF) | | Bodily Pain (BP) | | Vitality (VT) | | | Mental Health (MH) | | | |  |
| --- | --- | --- | --- | --- | --- | --- | --- | --- | --- | --- | --- | --- | --- | --- |
| A9A. Latent classes  + Physical Activity | Coefficient  (95% CI) | P-value | Coefficient (95% CI) | P-value | Coefficient  (95% CI) | P-value | Coefficient  (95% CI) | P-value | | Coefficient  (95% CI) | | P-value | |  |
| Healthy & active | Reference |  |  |  |  |  |  |  | |  | |  | |  |
| Healthy & inactive | 0.67 (-1.35; 2.73) | 0.509 | 0.44 (-0.75; 1.63) | 0.470 | 0.00 (-4.80; 4.80) | 1.000 | 0.00 (-3.55; 3.55) | 1.000 | | 0.35 (-3.78; 4.48) | | 0.868 | |  |
| At risk & active | -2.88 (-5.21; -0.54) | 0.016 | -1.01 (-2.33; 0.31) | 0.132 | 0.00 (-2.44; 2.44) | 1.000 | -2.50 (-5.18; 0.18) | 0.067 | | -1.49 (-3.60; 0.62) | | 0.167 | |  |
| At risk & inactive | -1.52 (-4.61; 1.58) | 0.336 | -1.85 (-3.17; -0.54) | 0.006 | -0.69 (-5.31; 3.92) | 0.768 | -2.50 (-5.72; 0.72) | 0.128 | | -2.70 (-5.20; -0.19) | | 0.035 | |  |
| Unhealthy & active | -1.60 (-3.99; 0.78) | 0.188 | -2.66 (-4.10; -1.23) | <0.001 | 0.00 (-2.98; 2.98) | 1.000 | 0.00 (-3.81;; 3.81) | 1.000 | | 0.20 (-2.64; 2.24) | | 0.871 | |  |
| Unhealthy & inactive | -4.79 (-9.43; -0.15) | 0.043 | -4.94 (-12.30; 3.41) | 0.187 | -6.15 (-19.87; 7.57) | 0.379 | -2.50 (-7.68; 2.68) | 0.344 | | -0.30 (-4.41; 3.80) | | 0.884 | |  |
| A9B. Metabolic Syndrome  + Physical Activity | | | | | | | | | | | | | |  |
| No MetS & active | Reference |  |  |  |  |  |  | |  | |  | |  | |
| No MetS & inactive | 1.19 (-1.13; 3.52) | 0.313 | 0.35 (-0.86; 1.56) | 0.567 | 0.00 (-4.44; 4.44) | 1.000 | 2.09 (-0.34; 4.52) | | 0.091 | | 0.24 (-3.10; 3.59) | | 0.886 | |
| MetS & active | -0.07 (-2.04; 2.18) | 0.946 | -0.93 (-2.35; 0.49) | 0.200 | 0.00 (-1.97; 1.97) | 1.000 | 0.86 (-1.51; 3.23) | | 0.477 | | 1.95 (0.27; 3.62) | | 0.023 | |
| MetS & inactive | -3.45 (-6.39; -0.50) | 0.022 | -2.91 (-4.66; -1.16) | 0.001 | -4.62 (-10.02; 0.78) | 0.094 | -3.76 (-7.40; -0.11) | | 0.044 | | -0.14 (-3.20; 2.92) | | 0.928 | |
| CI= Confidence interval;  Differences in median levels were analysed with quantile regression models; a coefficient of for example of -3.00 means that this group reached -3.00 less points in effect scores in the respective HRQoL domain, compared to the median of the reference group.  Adjustued for sex, age, education level study area, and HRQoL at SAPALDIA3 Categorized according to WHO Physical Activity Guidelines:  Inactive: <150 min of MPA and <75 VPA per week  Sufficiently active: >150 min of MPA or >75 VPA per week  Metabolic syndrome: Any three of the following values: Blood glucose ≥5.6mmol/L (Hba1C ≥5.7% in SAPALDIA4); waist circumference ≥ 94cm in men & ≥80cm in women; systolic blood pressure ≥130 or diastolic blood pressure ≥85; hdl <1.0mmol/L in men & <1.3mmol/L in women; triglycerides ≥1.7 mmol/L.  Cardiovascular diseases: Myocardial infarction, angina pectoris, heart insufficiency, claudicatio intermittens, stroke | | | | | | | | | | | | | | |

Additional Table 10. Cross-sectional associations of composite variable categories defined by latent classes (A10A) and metabolic syndrome (A10B), respectively, and physical activity status with health service utilization in the last 12 months, subjects not reporting any cardiovascular (SAPALDIA4)

| N=921 | Physician visit | | Hospital visit | |  |  |
| --- | --- | --- | --- | --- | --- | --- |
| A10A. Latent classes  + Physical Activity | Odds ratio  (95% CI) | P-value | Odds ratio (95% CI) | P-value |  |  |
| Healthy & active | Reference |  |  |  |  |  |
| Healthy & inactive | 1.02 (0.47; 2.24) | 0.873 | 1.15 (0.51; 2.59) | 0.732 | 1.00 (0.46; 2.19) | 0.999 |
| At risk & active | 0.93 (0.58; 1.50) | 0.847 | 0.90 (0.50; 1.61) | 0.719 | 0.94 (0.58; 1.52) | 0.795 |
| At risk & inactive | 1.01 (0.54; 1.89) | 0.860 | 1.90 (0.97; 3.72) | 0.062 | 0.97 (0.52; 1.82) | 0.934 |
| Unhealthy & active | 1.55 (0.86; 2.81) | 0.124 | 1.17 (0.62; 2.22) | 0.631 | 1.57 (0.86; 1.87) | 0.146 |
| Unhealthy & inactive | 0.84 (0.45; 1.57) | 0.626 | 2.10 (1.09; 4.04) | 0.026 | 0.85 (0.45; 1.60) | 0.611 |
| A10B. Metabolic syndrome (MetS)  + Physical activity | | | | |  |  |
| No MetS & active | Reference |  |  |  |  |  |
| No MetS & inactive | 0.82 (0.49; 1.37) | 0.443 | 2.01 (1.18; 3.44) | 0.011 | 0.78 (0.57; 1.31) | 0.351 |
| MetS & active | 1.36 (0.85; 2.18) | 0.194 | 1.33 (0.79; 2.21) | 0.281 | 1.31 (0.82; 2.11) | 0.258 |
| MetS & inactive | 1.18 (0.69; 2.02) | 0.548 | 1.91 (1.09; 3.37) | 0.024 | 1.16 (0.67; 2.01) | 0.600 |
| CI= Confidence interval;  Probabilities were calculated using logistic regression models for binary outcomes  Outcomes: 0 vs. ≥1  Adjustued Variables: Sex, age, education level and study area  Physical Activity Guidelines (WHO):  Inactive: <150 min of MPA and <75 VPA per week  Sufficiently active: >150 min of MPA or >75 VPA per week  Metabolic syndrome: Any three of the following values: Blood glucose ≥5.6mmol/L (Hba1C ≥5.7% in SAPALDIA4); waist circumference ≥ 94cm in men & ≥80cm in women; systolic blood pressure ≥130 or diastolic blood pressure ≥85; hdl <1.0mmol/L in men & <1.3mmol/L in women; triglycerides ≥1.7 mmol/L. | | | | | | |
